# Chemical Characteristics of Croatian Traditional *Istarski pršut* (PDO) Produced from Two Different Pig Genotypes

**DOI:** 10.3390/molecules26144140

**Published:** 2021-07-07

**Authors:** Marina Krvavica, Dario Lasić, Jasenka Gajdoš Kljusurić, Jelena Đugum, Špiro Janović, Srđan Milovac, Jasna Bošnir

**Affiliations:** 1 Department of Food Technology, Marko Marulic Polytehnic in Knin, Kralja Petra Krešimira 30, 22300 Knin, Croatia; mkrvavica@veleknin.hr; 2 Andrija Stampar Teaching Institute of Public Health, Mirogojska 16, 10000 Zagreb, Croatia; dario.lasic@stampar.hr (D.L.); srdan.milovac@stampar.hr (S.M.); jasna.bosnir@stampar.hr (J.B.); 3 Faculty of Food Technology and Biotechnology, University of Zagreb, Pierottijeva 6, 10000 Zagreb, Croatia; 4 Ministry of Agriculture, Ul. grada Vukovara 78, 10000 Zagreb, Croatia; jelena.dugum@mps.hr; 5 Department of Nursing, University North, Trg dr. Žarka Dolinara 1, 48000 Koprivnica, Croatia; spiro.janovic@unin.hr

**Keywords:** *Istarski pršut*, Duroc, dry-cured ham, amino acids, fatty acids, lipid oxidation

## Abstract

Chemical characteristics of raw and processed *Istarski pršut* (PDO) produced from two different pig genotypes were studied with special emphasis on amino and fatty acid composition and factors of lipid stability. Raw hams of Large White (LW)xLandrace (L), and (LWxL)xDuroc (D) pig genotypes were used in the study (20 hams of each genotype). All left raw hams from each carcass were processed in accordance with the PDO specification of *Istarski pršut*, and other half (the right ones) of LWxL)xD genotype were used for analyses of raw hams (fresh muscles). *Istarski pršut* was evaluated on the basis of the chemical parameters of the raw and matured lean ham. The process of dry curing significantly influenced the chemical properties of *Istarski pršut*. Despite the higher content of intramuscular fat and polyunsaturated fatty acids, the fat of (LWxL)xD ham was much more resistant to hydrolysis and oxidation, suggesting that fatty acid profile and other factors, also play a significant role. Significant differences between pig genotypes in the amino acid and fatty acid profiles were found. The analyzed *Istarski pršut* may be distinguished by prints of multivariate chemometric statistical analysis, based on their amino acid and fatty acid compositions.

## 1. Introduction

Due to their specific quality and organoleptic characteristics, four types of Croatian dry-cured ham (*Istarski*, *Krčki*, *Dalmatinski* and *Drniški pršut*) have been legally recognized as an exceptional contribution to the gastronomic culture of European Union and marked with the labels of Protected Designation of Origin (PDO) and Protected Geographical Indications (PGI). Among them, only *Istarski pršut* is marked with the PDO label, and its production from the beginning to the end of the process (including pig rearing) takes place in a geographical area defined by the PDO specification. *Istarski pršut* is a highly appreciated dry-cured ham, especially in Croatia and other regional countries, therefore the PDO label guarantees its specific quality and attributes expected by consumers. Compared with the other three types of hams mentioned, *Istarski pršut* is produced from pigs with a live weight over 160 kg. The hams are processed with pelvic bones and without skin and subcutaneous fat. Spices are used in the dry salting process, and the drying process is carried out without smoking [1]. The PDO specification of *Istarski pršut* also regulates the rearing of the animals. Only gilts and barrows (to avoid boar taint) of modern pig genotypes are allowed, except Pietrain (to avoid pale, soft and exudative–PSE meat). Animals must be at least 9 months old before slaughter.

It is well known that a variety of factors affect the final quality of dry-cured ham, but all of them are related to the characteristics of raw ham or processing technology. If the processing technology is standardized (as in the case of PDO products), the quality of the final dry-cured hams is primarily determined by the quality of the raw ham [2]. The quality of the raw ham depends mainly on factors related to the pig (breed, genes, sex, age, weight, diet) as well as on the pre/post-slaughter treatment (loading, transport, stunning, bleeding, dehairing, scalding, cooling) [2].

It is generally accepted that the meat of highly muscled pig genotypes, such as Pietrain (but also Belgian Landrace) are less suitable for the production of dry-cured ham due to the lower meat quality [3]. Hogs susceptible to porcine stress syndrome often develop post-mortem PSE meat, which is manifested by abnormally pale color, soft texture, and extremely low water- holding capacity of the meat. Low carcass fat content, particularly intramuscular fat (IMF) is also a common feature of meat from highly muscled swine genotypes. A large number of studies have reported a negative effect of PSE meat and a positive effect of IMF content on sensory characteristics of dry-cured meat products such as marbling, flavor, juiciness and tenderness [2,4,5,6,7,8,9]. The influence of pig genotype on the quality of raw ham destined for dry-cured ham production has also been widely studied [10,11]. Among the different pig genotypes that have been used to obtain the best quality of raw hams, the Duroc breed and especially its crossbreds frequently meet both muscle and fat quality criteria for dry-cured ham processing [2,12,13,14]. In addition, genes associated with the fatty acid composition and other important pork quality indicators in Duroc pigs have been recently identified and validated [15,16]. Although numerous studies have been conducted on the influence of genotype and, in particular, Duroc breed on the quality of different types of dry-cured ham, especially on its chemical profiles, only a few have been conducted on *Istarski pršut* [17,18]. Moreover, few studies have been published on the free amino acid content of *Istarski pršut* independent of the genotype effect [19,20], and there are no available data on the composition of total amino acids (free and peptide-bound) of *Istarski pršut*.

Lipids are responsible for many desirable properties (contributing to the improvement of taste, tenderness and juiciness) of meat and meat products [21]. However, they are also among the most chemically unstable components of meat, and are prone to degradation; especially in the processes of their hydrolysis and oxidation. Most authors recognize that the accumulation of free fatty acids (rich in unsaturated FAs) in the processes of lipid hydrolysis promotes their oxidation [22,23,24]. However, some consider that these two processes are independent or even that some free FAs have antioxidant properties and long-chain free FAs “protect” against oxidation [25]. In general, the oxidation processes can cause a non-microbial quality deterioration of meat and meat products, and their products have negative effects on the quality of meat and meat products [26,27]. However, in the case of dry-cured meat products, they also play an important role in the development of the typical flavor of the product, which is highly appreciated by consumers [28]. The profile and ratio of chemical compounds formed by lipid oxidation depends largely on the lipid profile of the animals (which depends on genotype, sex, age, fatness, weight, diet etc.), but also on other numerous post-mortem factors such as: processing methods, storage conditions, type of ingredient, and presence and concentrations of pro- or antioxidants [29]. All these numerous ante- and post-mortem factors affect the chemical profile of raw and processed meat. Although lipid oxidation has been intensively studied for decades, the mechanisms of lipid oxidation are not fully understood due to the complex reactions involved in this process and the different pathways and factors that influence it [30].

As the technology of *Istarski pršut* is very specific and unique compared to all other types of dry-cured ham around the world [17] (e.g., final trimming of raw ham without skin and subcutaneous fat tissue, specificities in the use of spices etc.), it is expected that the chemical profile of matured *Istarski pršut* will be significantly different from other ham varieties, regardless of the genotype of the pig. Because of these differences in processing technology, the influence of the Duroc breed on the chemical profile of *Istarski pršut* could be significantly different from that of other types of dry-cured ham.

Consequently, the aim of this study was to determine the differences between two pig genotypes often used in the production of Croatian dry-cured ham types, in chemical properties of *Istarski pršut*, such as chemical proximate composition, amino acid (AA) and fatty acid (FA) composition, as well as the influence of processing technology on the FA profile of mature hams. Particular emphases are placed on the FA composition of both raw and mature ham, and its influence on the level of lipolysis (acid value) and lipid oxidation (peroxide value and TBARS test) of *Istarski pršut* of both pig genotypes.

## 2. Results and Discussion

### 2.1. Chemical Properties

Proximate chemical analysis, NaCl content and pH of raw and matured ham are shown in Table 1.

In agreement with similar studies of other dry-cured hams [31,32], the influence of processing on the basic-chemical properties of *Istarski pršut* in this study was shown by a decrease in water content and an increase of dry matter content (proteins, IMF and NaCl) as well as an increase in pH, as expected [2]. Other authors, [33] stated that *Istarski pršut* has the following contents: water 37.9 to 41.0%, proteins 32.4 to 43.1%, fat 13.5 to 17.0% and NaCl 6.3 to 7.4%, and most of the results of this study fit into the indicated ranges, except for NaCl content which was higher (8.44%).

The differences between pig genotypes in chemical composition and NaCl content, as well as pH and weight loss during processing of *Istarski pršut* is presented in Table 2. All observed indicators, except protein content and pH, were strongly influenced by the genotypes (*p* < 0.01; *p* < 0.001) which is contrary to some authors [17,34], but also in agreement with others [35,36,37].

As expected, a significant influence of Duroc genotype on IMF content was found, which has long been considered one of the breed traits of Duroc [38] and has been confirmed by many authors [2,9,37,39,40]. Apart from the possible influence of Duroc genotype, the high content of IMF in (LWxL)xD of raw ham is certainly a consequence of the prolonged fattening [41] and the much higher final weights of the pigs [42].

Since IMF impedes dehydration, the higher water content and lower salt content in the (LWxL)xD hams could be due to the higher IMF content. The protein content in dry-cured ham depends on the degree of dryness and fat content [43].

In contrast to authors who found significantly lower weight loss of dry-cured ham of the Duroc genotype [39], these differences between the LWxL and (LWxL)xD hams were not significant (*p* > 0.05). In general, the weight loss of *Istarski pršut* determined in this study agrees with some studies that also found a weight loss of more than 40% [17,20]. The significantly higher weight loss of *Istarski pršut* compared to most other types of Mediterranean dry-cured hams is due to the unique shape of the ham with pelvic bones and without skin and subcutaneous fat, which exposes a larger surface area of the muscle tissue of the leg to drying [17,20]. Moreover, a significant difference in the weight loss of *Istarski pršut* was found between Pietrene and Duroc genotypes, but not between the Landrace and Duroc genotypes [17], which is consistent with the results of this study.

### 2.2. Amino Acid Composition

The composition of total amino acids (AA) of *Istarski pršut* is shown in Table 3. A total of 37.04 g AA/100 g sample was determined, of which the 9 essential (EAA) and 8 non-essential AA (NEAAs). Since the average amount of EAA and NEAA, regardless of genotype, was 18.29 and 18.75 g/100 g sample respectively (EAA/NEAA = 0.98), it can be said that dry-cured ham is an excellent source of biologically high-quality proteins, as it contains essential amino acids in appropriate ratios [44]. According to Zuo et al. [45], Chinese traditional dry-cured Jinhua ham has a slightly different AA profile; it contains a total of 27.67 mg AA/mL, of which the 16.20 mg/mL of EAA and 11.85 mg/mL of NEAA (EAA/NEAA = 1.37).

The differences between the genotypes in total AA, calculated per amount of sample, were not significant (*p* > 0.05), but calculated per amount of total proteins, they were significant (*p* < 0.01). The differences were significant only on NEAAs such as proline (*p* < 0.01), glycine (*p* < 0.05) and cysteine (*p* < 0.001) when calculated per amount of sample, but they were much larger when calculated per amount of total proteins. All EAAs, except of threonine, and five NEAAs (glutamic acid, proline, glycine, cysteine and tyrosine) differed significantly between genotypes (all of EAAs, except arginine, were higher in LWxL; all of NEAAs, except tyrosine, were higher in (LWxL)xD genotype). Consequently, the EAA/NEAA ratio was significantly lower in the (LWxL)xD genotype (*p* < 0.001). Since the content of free AAs is an indicator of proteolysis and increases with the progress of ham processing [5], much research has been conducted on their content in dry-cured ham, but not so much in total (free and peptide-bound) amino acids [45].

### 2.3. Fatty Acid Composition

The influence of processing technology on the FA composition of *Istarski pršut* is presented in Table 4, from which the considerable changes in FA composition of raw vs. matured ham, are visible.

One of the key changes in the ham tissues (beside dehydration, salt intake and proteolysis) affected by the dry-curing process, is lipid degradation (lipolysis) under the influence of lipolytic enzymes [2]. Many studies have shown those effects [32,46,47,48,49]. According to the results of this study, monounsaturated (MUFA) and saturated (SFA) fatty acids, were the main FAs found in the IMF of both, raw and matured ham (mostly due to oleic and palmitic FA contents). Together, they represent 93.44 % of total FAs in raw and 85.11 % in matured ham. Those results are close to the results of other studies [41,50,51]. Further, in raw and matured ham, a different number of FAs, 11 vs. 26, respectively were detected. Between the investigated groups, the differences of all FAs, except stearic (18:0), were significant (mostly *p* < 0.001), so myristic (14:0), palmitic (16:0), palmitoleic (16:1), oleic (18:1ω-9c), arachidonic (20:0; not detected in matured ham) and γ-linolenic acid (18:3ω-6), and SFA, MUFA and ω-9 as well, were higher in raw ham (*p* < 0.001). On the contrary, polyunsaturated (PUFA) were higher in raw ham (*p* < 0.001), and SFA and MUFA were higher in matured ham (*p* < 0.01 and *p* < 0.001, respectively). The ω-6 were found only in matured ham, while the content of ω-3 were significantly higher in the matured ham than in the raw ham (*p* < 0.001). For comparison, one study reported different results for Toscano ham. After 16 months of processing, they reported significant decrease of myristic (14:0), palmitic (16:0), palmitoleic (16:1), oleic (18:1ω-9) and cis-vaccenic (18:1ω-7) acid, and significant increase of arachidonic (20:4ω-6) acid [50]. They also reported a significant decrease of SFA and MUFA, and a significant increase in the ratio of ω-6/ω-3, but after 18 months of processing, they were no longer significantly different from raw ham. However, according to the results of Larrea et al. [52], PUFA, ω-3 and ω-6 increased in neutral (the most abundant) lipid fraction of matured Teruel ham, while in total lipids they were significantly lower in mature compared the raw Teruel ham, but they were decreased up to half of the process, after which they were gradually increased until the end of the process.

Many researchers reported significant impact of the length of processing on decrease of FAs in the neutral (triacylglycerols) and polar (phospholipids) lipid fractions of the IMF lipids, and their increase in the free FA fraction [47,50,53]. In the free FA fraction, particularly the MUFAs increase along the dry-curing process [51], although, besides the length of processing, the processing methods’ differences also have a significant impact [54]. Since the free FAs are prone to oxidation and reactions with other compounds, their quantity and ratio change at some ham ripening stage.

The differences between two pig genotypes in the FA profile of *Istarski pršut* are presented in Table 5.

The differences in FA composition of various dry-cured ham types are mainly the consequence of the pigs’ genetic features and differences in the rearing/feeding system. Recent research identified quantitative trait loci on swine chromosomes associated with stearic, oleic and SFA in Duroc breed [15]. Since the same feeding system was applied in both genetic groups of this study, the differences presented in Table 5 can be considered to be mainly the result of differences in the pig genotype [55,56]. The differences were significant for 17 of 27 FAs detected; and the most represented oleic (18:1ω-9c) were significantly higher in LWxL hams; stearic (18:0) and linoleic (18:2ω-6) acids were significantly higher in the (LWxL)xD hams, while the palmitic (16:0) was similar in both genotypes. On the contrary, Božac et al. [17] found no significant influence of Duroc genotype on the FA profile of *Istarski pršut*, except for higher content of myristic (14:0) acid. However, most studies found a significant effect of Duroc genotype on FA profile of dry-cured ham [8,14,18,47,56,57].

### 2.4. Lipolysis and Lipid Oxidation Indicators

The acid value (an indicator of the content of free FA), and peroxide value and TBARS assay (indicators of primary and secondary lipid oxidation) of *Istarski pršut* are presented in Table 6.

As can be seen, all the indicators were significantly different between the genotypes. According to the obtained results, despite the significantly higher IMF and PUFA in Duroc genotype, the IMF were much more resistant to hydrolysis and oxidation. This could be explained by the different influence of individual fatty acids (in total and free FA fraction) on fat stability, but probably the enzyme activities (mostly endogenous lipases and phospholipases) play an important role. The significantly lower peroxide value and TBARS in Duroc genotype could be explained with significantly lower acid value because, in general, fatty acids have been shown to oxidize faster in the free form than their glyceryl esters [24,29], not because of the length of their chains, but because of the number of bis-allylic positions, which cause the lipid peroxidation, to increase exponentially [58]. According to Cava et al. [51], free FA fraction largely increased throughout the ham ripening, and with it at the same time, the content of MUFAs largely increased from 34.7% to 40.9%, and PUFAs largely decreased from 45.1% to 20.5%, and SFAs increased from 28.3% to 38.6%. Also, some authors strongly suggest that the hydrolysis of phospholipids (which are composed of much more PUFA than triacylglycerols) during processing “protect” the long-chain PUFAs from oxidation, although the exact mechanism remains unknown [25,59]. Those reports could go towards explaining the results of this study. The higher content of NaCl in LWxL genotype could also be one of the promoters of lipolysis [53]. To better understand factors of lipolysis and fat oxidation, a correlation was made between FAs, IMF and NaCl contents and indicators of fat stability (Table 7). The results from Table 7 indicate, the higher the IMF content, the higher the content of stearic (18:0), linoleic (18:2ω-6), α-linolenic (18:3ω-3) and tetracosanoic acid (24:0), and the lower the content of elaidic (18:1ω-9t), arachidic (20:0), arachidonic (20:4ω-6) and heneicosanoic acid (20:0).

According to Ruiz et al. [6], the IMF content positively influenced the content of oleic acid (18:1ω-9c) and exhibited a negative relationship with linoleic (18:2ω-6) and arachidonic (20:4ω-6) acid. These authors explain that as the IMF increases, triacylglycerols which contain more oleic acid increase too; and because the content of phospholipid fraction which contains more PUFA is stable, the content of PUFA in total IMF relatively decreases. However, those results are in accordance with this study only regarding the linoleic (R = −0.51; *p* < 0.05) and arachidonic acid (R = −0.60; *p* < 0.01), since IMF content has not affected the content of oleic acid and neither the PUFA (Table 7). Further, the higher the content of IMF, the lower the contents of UnidFA, MUFA, ω-9 and NaCl (Table 7). Since higher content of NaCl has not affected the AV nor PV and TBARS; increased content of NaCl was not shown as a promoter of lipolysis and lipid oxidation. Most research points to salt as a promoter of lipolysis and oxidation [53,60,61,62], but there are those who do not find such an influence [63,64].

The higher AV and PV were positively associated with the contents of 10-heptadecanoic (17:1), eicosanoic (20:0) and heneicosanoic (21:0) acid and negatively with the content of stearic acid (18:0). The higher the stearic acid content, the more stable the fat was, since AV and PV were significantly lower (*p* < 0.001 and *p* < 0.05, respectively) and the correlation with TBARS values was also very close to the statistical significance (R = 0.40). However, only the content of two FAs affected TBARS, palmitoleic acid (16:1) positively, and tetracosanoic acid (24:0) negatively. The higher the contents of SFA and PUFA, the lower the AV (*p* < 0.01), but not the PV and TBARS. In addition, the higher the contents of MUFA and UFA, the higher the AV (*p* < 0.001 and *p* < 0.01, respectively), but not the PV and TBARS. Also, the TBARS and PV were in highly positive correlation (R = 0.95; *p* < 0.001).

Additionally, the correlation between the lipolysis/lipid oxidation indicators, within the (LWxL)xD genotype were highly positive between PV and AV (R = 0.87; *p* < 0.001) as well as PV and TBARS (R = 0.94; *p* < 0.001), while within the LWxL genotype were highly positive only between PV and TBARS (R = 0.95; *p* < 0.001).

### 2.5. PCA Analysis

**Figure 1:**
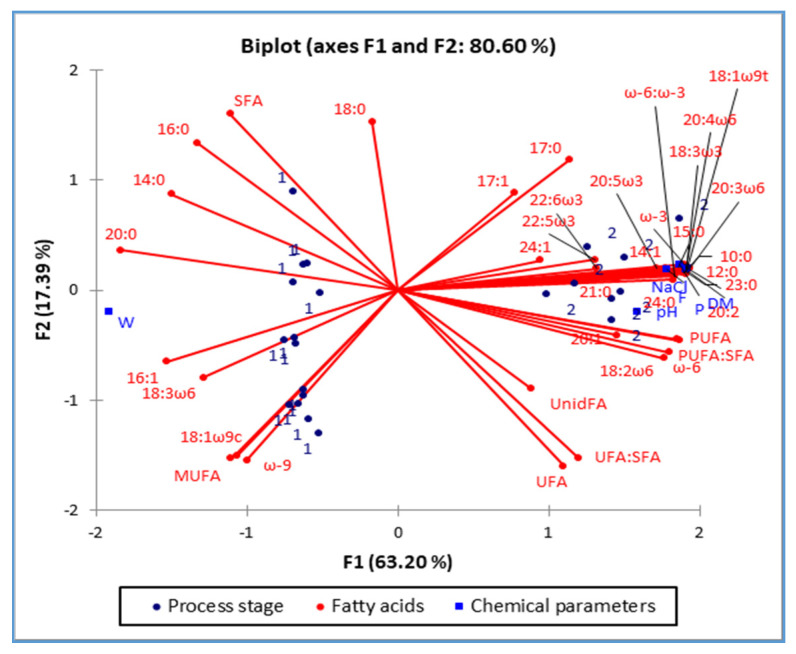
Score plot of the first two principal components (PC1 and PC2), of the principal component analysis, showing variance based on the fatty acid composition of ham from two different process stages, raw ham (1) and matured ham (2).

**Figure 2:**
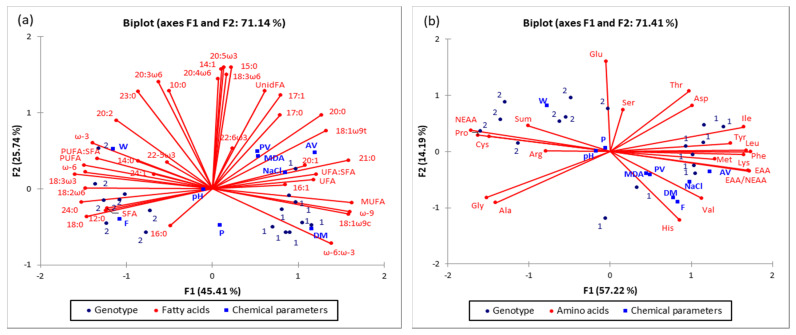
Score plot of the first two principal components (PC1 and PC2), of the principal component analysis, showing variance based on the (**a**) fatty acid composition and (**b**) amino acid composition of matured *Istarski pršut* of two different genotypes, LWxL (1) & (LWxL)xD (2).

The score plots of the Principal component analysis are presented in Figure 1 and Figure 2. Figure 1 shows the grouping and changes in the FA profile of the raw and matured ham, while Figure 2 shows differences in the FA (Figure 2a) and AA profiles (Figure 2b) of two different genotypes of matured ham.

The application of multivariate analysis in meat science, especially for pork meat, becomes an indispensable tool in the scientific investigation. For example, some authors related the elemental composition of pork meat from conventional and animal welfare farms by inductively coupled plasma-optical emission spectrometry (ICP-OES) and ICP-mass spectrometry (ICP-MS) and their authentication via multivariate chemometric analysis [65], while others used multivariate tools in investigating authentication of organic pork and identification of geographical origins of pork in four regions of China by combined analysis of stable isotopes and multi-elements [66]. In this study, multivariate tool, the PCA was used to identify which fatty acid (FA) or amino acid (AA) dominates in different genotypes of pork used in processing of *Istarski pršut*, as well as to investigate FAs and AAs in hams from two different process stages (raw and matured).

Score plots (Figure 1 and Figure 2) give an insight into a system in which a significant number of variables were observed simultaneously. So, the processing influence on the FA composition of the raw ham is presented in Figure 1. First two principal components (PC1 & PC2) explain 80.6% of the variability of all observed data. Also, it should be noted that the raw ham (Figure 1, marked as “1” and positioned in the 2nd and 3rd quadrant) has a significantly higher content of water (which is seen by occupying the left part of the plot). In the left part of the plot are also positioned FAs which had not changed significantly during the processing (and those FAs are: 14:0; 16:0; 16:1; 18:0; 20:0; SFA; MUFA; ω-9; 18:3ω6 and 18:3ω9c).

By observing of two genotypes of ham, it was found that the first two PCs contributed with 71.41%, regardless if FAs (Figure 2a) or AAs (Figure 2b) are observed. Such high sum of the first two PCs indicates that over 71% of all interactions in the observed data set, can be explained based on the observed genotype, basic chemical composition & FA and AA contents. The investigated genotypes ((LWxL = 1 & (LWxL)xD = 2) were moved away and positioned into different quadrants. Ham of the LWxL genotype (1) is positioned in the right part of the chart, while the matured ham of the other observed genotype (2 = (LWxL)xD genotype) is positioned in the left part of the chart, for both observed concentrations of FAs and AAs. Such spreading in the Bi-plot and grouping based on the genotype indicates the qualitative differentiation of the samples (matured *Istarski pršut* produced from two different pork genotypes). Additional multivariate tools would be needed for quantitative prediction of certain FA and or AA if the genotype is known or vice versa. It is Figure 2a that shows that the content of Alanine (Ala), Glycine (Gly) will differ based on the observed genotype, but not significantly (they are positioned in the 3rd quadrant indicating no significant differences of Ala & Gly in the samples positioned in adjacent quadrants, such as genotype 2 (positioned in the 2nd quadrant) or the majority of genotype 1, positioned in 4th quadrant). However, position of AA as Arginine (Arg), Proline (Pro) and Cysteine (Cys), in the same quadrant where are grouped the hams of (LWxL)xD genotype, indicate that should be expected significantly different values regarding the observed genotype 2.

The PCA plots related to the values in Table 1, Table 2, Table 3, Table 4, Table 5 and Table 6 confirm the significance of the changes in the chemical properties of raw and matured ham (Table 1) and different pig genotypes (Table 2) with the AA composition of *Istarski pršut*, depending on genotype (Table 3), as well as the changes in FA composition, depending on processing (Table 4) and genotype (Table 5), and changes in indicators of lipolysis and lipid oxidation depending on genotype (Table 6). It can be assumed that PCA application cannot provide empirical results to quantify the effects or clarify variations between processing and genotypes like other statistical approaches. However, it helps to understand better and substantiate relationships between a variety of variables affecting meat quality by reducing the data and visualizing them using PCA.

## 3. Materials and Methods

### 3.1. Raw Ham Selection and Processing

Raw ham (purchased at the market) selection and shaping: The raw hams used in this study were obtained from the 20 pigs of two different genotypes (5 gilts and 5 barrows per genotype), raised in the same pen (same farm) under the same conditions and fed ad libitum with the same commercial feed. Genetic background of the animals (10 of each genotype) was as follows:

- Genotype 1 (LWxL): ♀ Large White (LW) × ♂ Swedish Landrace (L) and
- Genotype 2 (LWxL)xD: ♀ (LWxL) × ♂ Duroc (D).

The animals were 12 months old at slaughter when they reached an average live slaughter weight of 182.9 kg for genotype 1 and 186.3 kg for genotype 2. Average hot carcass weights were 151.2 kg and 154.4 kg, and killing out percentage were 82.64% and 82.89%, for genotype 1 and genotype 2, respectively. After slaughtering, in accordance with the industry-accepted procedure, the hams were removed from the carcasses according to the Istrian manner (PDO specification). The hams were separated between the last lumbal (*v. lumbales*) and first sacral vertebra (*v. sacrales*). The pelvic bones such as *os ilium*, *os ischii* and *os pubis*, were left in the ham and only the sacrum (*os sacrum*) and caudal vertebra (*v. caudales*) were removed. The leg was cut at the ankle (*a. tarsi*) so that in connection with the tibia and fibula remains the proximal row (*talus* and *calcaneus*) of the ankle bones. On the lateral and medial side of the ham, the skin and subcutaneous adipose tissue were removed to a height of 10–15 cm proximal to the ankle. The hams treated in this way are characteristically long and closed surfaces. After shaping the raw ham, weights of genotype 1 and genotype 2 were 14.58 kg and 14.91 kg, respectively. All left hams from each carcass were subjected to processing, and the right ones were used to take samples of raw ham (fresh muscles).

Ham processing (20 left hams): Immediately before salting, the hams were vigorously massaged by hand to remove residual blood, especially from the femoral artery (*a. femoralis*) and other visibly bloody areas. In the process of dry salting, hams were salted with a mixture of coarse and ground sea salt (in a 70:30 ratio) and spices (4.5% per kg of NaCl) such as ground black pepper, garlic and laurel. Salting was done by hand, firmly rubbing the dry-salt mixture (0.6–0.7 kg per ham) on the surface of the hams, after which they were left on the shelves for 21 days at a temperature of 2–5 °C. During the 21 days of salting period, the hams were rotated twice. After the salting phase, the hams were washed with cold water, to remove excess salt, and left to drain for 24 h. After draining, hams were rubbed with ground pepper and then subjected to the drying process. The drying process was carried out under natural climate conditions (the drying rooms were exposed to dominant winds) for 6 months at the temperature of 12–15 °C and relative humidity of 65–75%. After the salting and drying phases, when the hams lost about 35% of their initial weight, they were moved to the ripening phase for the next 9 months, under the stable microclimate at a temperature up to 18 °C and relative humidity of 70–75%.

After 15 months of processing, the mature hams of genotype 1 and genotype 2 were weighing 8.58 kg and 8.89 kg, respectively. At the beginning and the end of the process the pH of the *m. semimembranosus* was measured using the pH meter CPC-501 ELMETRON (ELMETRON ©, Zabrze, Poland) equipped with a combined puncture pH electrode, OSH 12-01.

### 3.2. Sampling

A longitudinal section from *tuber ishiadicum* to *tuber calcanei* of the hams, both raw and matured was made, and the samples (approximate 200 g in weight) composed mainly of *m. semimembranosus*, *m. semitendinosus* and *m. bicep femoris* muscles were taken (all visible fat and connective tissue from the samples were removed). Samples were individually vacuum packaged, coded, frozen and stored at −20 °C until analysis. By the end of the first week after freezing, both raw and matured samples were analyzed (to avoid chemical changes caused by storage). Before analysis, the samples were thawed for 24 h at 4 °C and homogenized.

### 3.3. Chemical Analyses

Proximate chemical analysis and fatty acid analysis were carried out on both raw and matured samples of lean ham. Determination of amino acids and lipolysis and lipid oxidation indicators were carried out only on matured samples of lean ham. Only the samples of raw and matured hams of (LWxL)xD genotype were used to assess the effect of processing on the proximate chemical composition and FA composition of *Istarski pršut*.

Moisture, fat and protein contents, as well as sodium chloride content were determined according to methods recommended by the AOAC [67]. Results were expressed as wt% of sample.

The AA content was determined according to Holló et al. [68] in an automatic amino acid analyzer (INGOS AAA 400, INGOS Ltd., Prague, Czech Republic) of the previously hydrolyzed proteins using reusable Pyrex hydrolysis tubes. In case of the AA containing sulfur performic acid, oxidation was made before hydrolysis according to Csapó et al. [69]. Samples are filtered and stored at −25 °C until the analysis by ion exchange column chromatography. The determination of amino acids was performed with post column derivatization by ninhydrin with photometric detection at 570 nm for all amino acids and 440 nm for proline. Results were expressed as g AA/100 g sample as well as g AA/100 g proteins.

Analysis of FA methyl esters was determined by gas chromatography according to ISO 12966-2 method [70]. Each sample’s fat was extracted using solvent petroleum ether (User Manual Soxtec System 2047 SoxCap) according to ISO 1443 method [71]. All lipid extracts were evaporated to dryness with nitrogen stream at 35 °C and stored at −18 °C until preparation of their fatty acid methyl esters (FAMEs). Lipids were transesterified under sequential alkali- and acid-catalyzed conditions by heating in methanol solution. After esterification, FAMEs were isolated by extraction with isooctane according to ISO 12966-2 method [70] and stored at −18 °C until chromatographic analysis. Separation and quantification of the FAMEs was carried out using a gas chromatograph, GC-Shimadzu, Model: GC-2010 Plus (Shimadzu Corporation, Duisburg, Germany) equipped with a flame ionization detector and an automatic sample injector AOC-5000 Shimadzu, and using an Agilent J & W DB 23-fused silica capillary column (60 m, 0.25 mm i.d., 0.25 μm film thickness). The chromatographic conditions were as follows: initial column temperature 60 °C held for 1 min, then increased at 7 °C/min to 215 °C and held 30 min. The injector and detector were maintained at 250 and 260 °C, respectively. Nitrogen was used as carrier gas at a constant flow-rate of 1.50 mL/min, with the column head pressure set at 179.9 kPa. The split ratio was 1:80 and 1 μL of the solution was injected. Individual FAMEs were identified by comparing their retention times with those of authenticated standards. Results are expressed as a percentage (%) of particular fatty acid on total fatty acids.

Acid value (AV), as an equivalent of the amount of free fatty acids, was used as an indicator of lipolysis. The acid value was determined according to ISO 660 method [72] and expressed as mg KOH/g fat.

Level of lipid oxidation was assessed by the determination of peroxide value (primary oxidation) and by the thiobarbituric acid assay (secondary oxidation). Peroxide value (PV) was determined according to the method recommended by AOAC [73], and expressed as meq/kg fat. Thiobarbituric acid (TBA) assay was conducted according to Lemon [74]. Absorbance at 538 nm was measured by a SPECORD 200 spectrophotometer (Analytic Jena AG, Germany). A calibration curve was developed using 0, 0.01, 0.02, 0.03, 0.04 and 0.05 μmol of malondialdehyde (MDA). TBARS values were expressed as mg of MDA equivalents/kg sample.

All the analyses were done in duplicate, except for AV, PV and TBARS, which were done in three replicates of each sample, and the average score for each sample was used for statistical analysis.

### 3.4. Statistical Analysis

Discriminant analysis and correlations were performed for all data collected. The categorial variables were the pork genotypes (coded as 1 (LWxL) & 2 (LWxL)xD), respectively. To determine the influence of pork genotype and processing on the scale factor of different factors and to create sample grouping, principal component analysis (PCA) was performed using XLSTAT 2016 software (Addinsoft, Paris, France). PCA was applied as a well-known technique for tracking and detecting similarities and/or differences in multivariate processes because it allows for the assessment of variability through dimensionality reduction. PCA was used to track overall process variability in amino-acid and fatty acid composition for two genotypes, as well as for the process stage.

## 4. Conclusions

From the obtained results, it can be concluded that most of the chemical properties of raw and matured *Istarski pršut* were significantly influenced by pig genotype and processing. The processing weight loss, and pH and protein content of *Istarski pršut* between the pig genotypes were not significantly different. On the contrary, the dry matter (content of water), intramuscular fat (IMF) and NaCl, and the profiles of AA and FA and lipolysis and the lipid stability of *Istarski pršut* were significantly different. Although the contents of intramuscular fat and polyunsaturated fatty acids were higher, the intramuscular fat of *Istarski pršut* of the Duroc genotype was much more resistant to hydrolysis and oxidation, suggesting that other factors also play a significant role. The assumption that the hydrolysis of phospholipids (which are composed of much more PUFA than triacylglycerols) during processing “protect” the long-chain polyunsaturated fatty acids from oxidation might be supported by the differences between genotypes in the fatty acid composition of *Istarski pršut* and significantly higher content of polyunsaturated fatty acids of Duroc genotype (*p* < 0.001). Multivariate chemometrics efficiently separated the studied samples based on the processing and genotype. Conclusively, the analyzed *Istarski pršut* can be distinguished by elemental fingerprints via multivariate chemometric statistical analysis based on their amino acid and fatty acid composition of the row ham genotypes.

## Figures and Tables

**Table 1:** Influence of processing on basic-chemical properties (means ± standard error) of raw and matured *Istarski pršut* of (LWxL)xD genotype.

Indicators	Raw Ham	Matured Ham	*p*
Dry matter (DM), %	27.62 ± 1.42	57.86 ± 2.26	**0.000**
Water (W), %	72.38 ± 1.42	42.14 ± 2.26	**0.000**
Protein (P), %	23.02 ± 0.55	37.70 ± 2.48	**0.000**
Fat (IMF), %	4.18 ± 1.42	16.12 ± 2.60	**0.000**
NaCl, %	-	8.29 ± 0.78	-
pH	5.77 ± 0.18	6.18 ± 0.28	**0.000**

The values in bold are significant.

**Table 2:** Differences between pig genotypes in basic-chemical properties (means ± standard error) of matured *Istarski pršut*.

Indicators	LWxL	(LWxL)xD	*p*
Dry matter (DM), %	63.00 ± 1.57	57.86 ± 2.26	**0.000**
Water (W), %	37.01 ± 1.57	42.14 ± 2.26	**0.000**
Protein (P), %	38.13 ± 3.51	37.70 ± 2.48	0.311
Fat (IMF), %	11.76 ± 2.17	16.12 ± 2.60	**0.000**
NaCl, %	9.04 ± 0.38	8.29 ± 0.78	**0.002**
pH	6.13 ± 0.18	6.18 ± 0.28	0.324
Processing weight loss, %	41.36 ± 3.48	40.48 ± 2.26	0.254

LW—Large White; L—Landrace; D—Duroc; The values in bold are significant.

**Table 3:** Differences between pig genotypes in the AA composition (means ± standard error) of matured *Istarski pršut*.

Amino Acid (AA)	gAA/100 g Sample			gAA/100 g Protein		
	LWxL	(LWxL)xD	*p*	LWxL	(LWxL)xD	*p*
Threonine (Thr)	1.72 ± 0.11	1.74 ± 0.18	0.392	4.62 ± 0.11	4.59 ± 0.04	0.226
Valine (Val)	2.02 ± 0.12	1.95 ± 0.21	0.168	5.40 ± 0.04	5.13 ± 0.19	**0.000**
Methionine (Met)	1.03 ± 0.07	1.02 ± 0.12	0.385	2.75 ± 0.05	2.68 ± 0.07	**0.006**
Isoleucine (Ile)	1.80 ± 0.13	1.76 ± 0.21	0.308	4.81 ± 0.12	4.64 ± 0.07	**0.001**
Leucine (Leu)	3.01 ± 0.20	2.95 ± 0.35	0.327	8.05 ± 0.11	7.78 ± 0.15	**0.000**
Phenylalanine (Phe)	1.55 ± 0.11	1.51 ± 0.18	0.297	4.15 ± 0.08	3.98 ± 0.07	**0.000**
Histidine (His)	1.68 ± 0.11	1.59 ± 0.18	0.083	4.51 ± 0.13	4.19 ± 0.16	**0.000**
Lysine (Lys)	3.31 ± 0.23	3.24 ± 0.42	0.323	8.86 ± 0.17	8.53 ± 0.23	**0.001**
Arginine (Arg)	2.26 ± 0.15	2.41 ± 0.30	0.089	6.07 ± 0.23	6.36 ± 0.35	**0.021**
**Total EAA**	18.38 ± 1.19	18.16 ± 2.08	0.386	49.24 ± 0.45	47.85 ± 0.62	**0.000**
Aspartic acid (Asp)	3.53 ± 0.24	3.57 ± 0.38	0.398	9.46 ± 0.09	9.41 ± 0.08	0.116
Serine (Ser)	1.49 ± 0.11	1.52 ± 0.19	0.314	3.97 ± 0.06	4.00 ± 0.13	0.278
Glutamic acid (Glu)	5.97 ± 0.46	6.25 ± 0.71	0.159	15.99 ± 0.47	16.45 ± 0.23	**0.011**
Proline (Pro)	1.55 ± 0.10	1.75 ± 0.16	**0.003**	4.15 ± 0.19	4.63 ± 0.24	**0.000**
Glycine (Gly)	1.77 ± 0.20	1.96 ± 0.15	**0.021**	4.75 ± 0.58	5.19 ± 0.43	**0.047**
Alanine (Ala)	2.22 ± 0.33	2.22 ± 0.21	0.494	5.70 ± 0.25	5.85 ± 0.14	0.073
Cysteine (Cys)	0.33 ± 0.03	0.41 ± 0.03	**0.000**	0.86 ± 0.10	1.09 ± 0.10	**0.000**
Tyrosine (Tyr)	1.57 ± 0.12	1.54 ± 0.16	0.293	4.19 ± 0.09	4.05 ± 0.08	**0.001**
**Total NEAA**	18.43 ± 1.15	19.20 ± 1.90	0.144	49.09 ± 0.45	50.66 ± 0.63	**0.000**
**EAA/NEAA**	1.00 ± 0.03	0.94 ± 0.02	**0.000**	1.00 ± 0.02	0.94 ± 0.02	**0.000**
**Total AA**	36.81 ± 2.29	37.36 ± 3.97	0.354	98.33 ± 0.14	98.51 ± 0.11	**0.003**
NH_3_	0.61 ± 0.04	0.60 ± 0.06	0.254	1.65 ± 0.08	1.58 ± 0.05	**0.022**
N% × 6.25	38.33 ± 3.87	37.60 ± 2.38	0.622	-	-	-

LW—Large White; L—Landrace; D—Duroc; EAA—Essential AA; NEAA—Non-essential AA; The values in bold are significant.

**Table 4:** Influence of processing on the FA composition (means ± standard error) of raw and matured *Istarski pršut* of (LWxL)xD genotype (% of FAMEs).

Fatty Acid	Raw Ham	Matured Ham	*p*
C10:0	0.00	0.08 ± 0.01	**0.000**
C12:0	0.00	0.08 ± 0.01	**0.000**
C14:0	1.94 ± 0.33	1.40 ± 0.10	**0.000**
C14:1	0.00	0.02 ± 0.01	**0.000**
C15:0	0.00	0.05 ± 0.02	**0.000**
C16:0	28.14 ± 3.19	24.22 ± 0.77	**0.001**
C16:1	3.81 ± 0.54	2.53 ± 0.30	**0.000**
C17:0	0.20 ± 0.06	0.28 ± 0.05	**0.002**
C17:1	0.18 ± 0.05	0.22 ± 0.04	**0.027**
C18:0	13.79 ± 1.72	13.72 ± 0.76	0.457
C18:1ω-9t	0.00	0.29 ± 0.03	**0.000**
C18:1ω-9c	44.64 ± 2.77	41.11 ± 0.79	**0.001**
C18:2ω-6	5.90 ± 2.08	11.84 ± 1.37	**0.000**
C18:3ω-3	0.24 ± 0.11	0.60 ± 0.08	**0.000**
C18:3ω-6	0.00	0.05 ± 0.02	**0.000**
C20:0	0.20 ± 0.05	0.00	**0.000**
C20:1	0.54 ± 0.16	0.88 ± 0.14	**0.000**
C20:2	0.00	0.62 ± 0.08	**0.000**
C20:3ω-6	0.00	0.18 ± 0.02	**0.000**
C20:4ω-6	0.00	0.72 ± 0.18	**0.000**
C20:5ω-3	0.00	0.02 ± 0.01	**0.000**
C21:0	0.00	0.02 ± 0.01	**0.000**
C22:5ω-3	0.00	0.20 ± 0.24	**0.001**
C22:6ω-3	0.00	0.02 ± 0.02	**0.000**
C23:0	0.00	0.11 ± 0.01	**0.000**
C24:0	0.00	0.03 ± 0.01	**0.000**
C24:1	0.00	0.09 ± 0.18	**0.029**
UnidFA	0.39 ± 0.34	0.64 ± 0.12	**0.029**
SFA	44.27 ± 4.73	39.98 ± 0.98	**0.010**
MUFA	49.17 ± 2.97	45.13 ± 0.99	**0.000**
PUFA	6.14 ± 2.17	14.25 ± 1.57	**0.000**
UFA	55.30 ± 4.56	59.39 ± 1.01	**0.011**
PUFA/SFA	0.14 ± 0.05	0.36 ± 0.05	**0.000**
UFA/SFA	1.27 ± 0.21	1.49 ± 0.06	**0.004**
ω-9	44.64 ± 2.77	41.40 ± 0.80	**0.002**
ω-6	5.90 ± 0.52	12.79 ± 1.39	**0.000**
ω-3	0.24 ± 0.11	0.84 ± 0.26	**0.000**
ω-6/ω-3	23.54 ± 2.39	15.98 ± 3.05	0.001

UnidFA—Unidentified fatty acids (most likely long-chain PUFA); SFA—Saturated fatty acids; MUFA—Mono-unsaturated fatty acids; PUFA—Polyunsaturated fatty acids; UFA—Unsaturated fatty acids; The values in bold are significant.

**Table 5:** Differences between pig genotypes in fatty acid properties (means ± standard error) of matured *Istarski pršut*.

Fatty Acid	LWxL	(LWxL)xD	*p*
C10:0	0.07 ± 0.02	0.08 ± 0.01	0.124
C12:0	0.07 ± 0.01	0.08 ± 0.01	**0.000**
C14:0	1.29 ± 0.13	1.40 ± 0.10	**0.032**
C14:1	0.03 ± 0.06	0.02 ± 0.01	0.249
C15:0	0.09 ± 0.11	0.05 ± 0.02	0.173
C16:0	23.55 ± 1.16	24.22 ± 0.77	0.086
C16:1	2.84 ± 0.27	2.53 ± 0.30	**0.014**
C17:0	0.40 ± 0.13	0.28 ± 0.05	**0.009**
C17:1	0.37 ± 0.15	0.22 ± 0.04	**0.008**
C18:0	11.23 ± 0.47	13.72 ± 0.76	**0.000**
C18:1ω-9t	0.46 ± 0.07	0.29 ± 0.03	**0.000**
C18:1ω-9c	46.42 ± 1.55	41.11 ± 0.79	**0.000**
C18:2ω-6	8.48 ± 1.24	11.84 ± 1.37	**0.000**
C18:3ω-3	0.17 ± 0.08	0.60 ± 0.08	**0.000**
C18:3ω-6	0.06 ± 0.06	0.05 ± 0.02	0.263
C20:0	0.23 ± 0.10	0.00	**0.000**
C20:1	1.04 ± 0.09	0.88 ± 0.14	**0.003**
C20:2	0.48 ± 0.12	0.62 ± 0.08	**0.006**
C20:3ω-6	0.16 ± 0.06	0.18 ± 0.02	0.134
C20:4ω-6	0.82 ± 0.25	0.72 ± 0.18	0.176
C20:5ω-3	0.04 ± 0.06	0.02 ± 0.01	0.231
C21:0	0.39 ± 0.07	0.02 ± 0.01	**0.000**
C22:5ω-3	0.12 ± 0.04	0.20 ± 0.24	0.145
C22:6ω-3	0.03 ± 0.01	0.02 ± 0.02	0.169
C23:0	0.08 ± 0.05	0.11 ± 0.01	**0.033**
C24:0	0.00	0.03 ± 0.01	**0.000**
C24:1	0.00	0.09 ± 0.18	**0.000**
UnidFA	0.89 ± 0.31	0.64 ± 0.12	**0.022**
SFA	37.39 ± 1.27	39.98 ± 0.98	**0.000**
MUFA	51.16 ± 1.29	45.13 ± 0.99	**0.000**
PUFA	10.35 ± 1.53	14.25 ± 1.57	**0.000**
UFA	61.51 ± 1.00	59.39 ± 1.01	**0.000**
PUFA/SFA	0.28 ± 0.05	0.36 ± 0.05	**0.001**
UFA/SFA	1.65 ± 0.08	1.49 ± 0.06	**0.000**
ω-9	46.87 ± 1.51	41.40 ± 0.80	**0.000**
ω-6	9.52 ± 1.38	12.79 ± 1.39	**0.000**
ω-3	0.36 ± 0.16	0.84 ± 0.26	**0.000**
ω-6/ω-3	29.24 ± 8.73	15.98 ± 3.05	**0.000**

LW—Large White; L—Landrace; D—Duroc; UnidFA—Unidentified fatty acids (most likely long-chain PUFA); SFA–Saturated fatty acids; MUFA—Mono-unsaturated fatty acids; PUFA—Polyunsaturated fatty acids; UFA—Unsaturated fatty acids; The values in bold are significant.

**Table 6:** Differences between pig genotypes in the indicators of lipolysis and lipid oxidation (means ± standard error) of matured *Istarski pršut*.

Indicators	LWxL	(LWxL)xD	*p*
Acid value (AV), KOH/g fat	38.50 ± 8.46	22.20 ± 5.16	**0.000**
Peroxide value (PV), meq/kg fat	17.80 ± 8.09	11.10 ± 7.45	**0.035**
TBARS, mg MDA/kg sample	0.42 ± 0.19	0.28 ± 0.12	**0.037**

LW—Large White; L—Landrace; D—Duroc; The values in bold are significant.

**Table 7:** Correlation (Pearson) matrix between individual FAs and lipolysis/lipid oxidation indicators of matured *Istarski pršut*, irrespective to genotype.

Variables	IMF	AV	PV	TBARS
C10:0	0.03	−0.11	0.06	0.10
C12:0	0.38	−0.67 **	−0.34	−0.34
C14:0	0.20	−0.39	−0.23	−0.06
C14:1	0.24	0.26	0.36	0.36
C15:0	0.30	0.34	0.40	0.40
C16:0	0.08	−0.29	−0.24	−0.12
C16:1	−0.40	0.25	0.36	0.46 *
C17:0	**−0.57**	0.46 *	0.32	0.32
C17:1	−0.43	0.54 *	0.44 *	0.43
C18:0	0.61 **	−0.77 ***	−0.44 *	−0.40
C18:1ω-9t	−0.70 ***	0.77 ***	0.38	0.41
C18:1ω-9c	−0.42	0.70 ***	0.37	0.32
C18:2ω-6	0.51 *	−0.65 **	−0.39	−0.42
C18:3ω-3	0.60 **	−0.71 ***	−0.37	−0.34
C18:3ω-6	−0.21	0.24	0.27	0.27
C20:0	−0.52 *	0.78 ***	0.50 *	0.43
C20:1	−0.28	0.70 ***	0.18	0.11
C20:2	0.33	−0.28	−0.17	−0.23
C20:3ω-6	−0.24	−0.08	0.07	0.08
C20:4ω-6	−0.60 **	0.31	0.27	0.30
C20:5ω-3	−0.28	0.28	0.35	0.32
C21:0	−0.61 **	0.81 ***	0.46 *	0.40
C22:5ω-3	−0.28	−0.16	−0.05	0.01
C22:6ω-3	−0.34	0.23	0.29	0.28
C23:0	−0.20	−0.17	0.02	−0.01
C24:0	0.66 **	−0.69 **	−0.40	−0.49 *
C24:1	−0.15	−0.30	−0.12	−0.05
UnidFA	−0.62 **	0.50 *	0.23	0.21
SFA	0.41	−0.63 **	−0.39	−0.28
MUFA	−0.48 *	0.73 ***	0.41	0.37
PUFA	0.42	−0.60 **	−0.34	−0.35
UFA	−0.36	0.65 **	0.36	0.24
PUFA/SFA	0.35	−0.51 *	−0.28	−0.32
UFA/SFA	−0.39	0.62 **	0.37	0.25
ω-6	0.43	−0.61 **	−0.36	−0.38
ω-3	0.23	−0.54 *	−0.22	−0.17
ω-6/ω-3	−0.29	0.53 *	0.15	0.09
ω-9	−0.44 *	0.71 ***	0.37	0.32
IMF	1.00	−0.48*	−0.18	−0.29
AV	−0.48 *	1.00	0.34	0.27
PV	−0.18	0.34	1.00	0.95 ***
TBARS	−0.29	0.27	0.95 ***	1.00
NaCl	−0.56 *	0.16	0.14	0.26

IMF—Intramuscular fat; AV—Acid value; PV—Peroxide value; TBARS—Thiobarbituric acid reactive substances assay; UnidFA—Unidentified fatty acids (most likely long-chain PUFA); SFA—Saturated fatty acids; MUFA—Mono-unsaturated fatty acids; PUFA—Polyunsaturated fatty acids; UFA—Unsaturated fatty acids; The values in bold are different from 0 (* *p* < 0.05; ** *p* < 0.01; *** *p* < 0.001).

## Data Availability

The data presented in this study are available on request from the corresponding author. The data are not publicly available due to the project agreement.

## Sample Availability

Samples of the compounds are not available from the authors.
